# Impaired Replication Stress Response in Cells from Immunodeficiency Patients Carrying Cernunnos/XLF Mutations

**DOI:** 10.1371/journal.pone.0004516

**Published:** 2009-02-18

**Authors:** Michal Schwartz, Yifat S. Oren, Assaf C. Bester, Ayelet Rahat, Ruthy Sfez, Shlomo Yitzchaik, Jean-Pierre de Villartay, Batsheva Kerem

**Affiliations:** 1 Department of Genetics, The Life Sciences Institute, The Hebrew University, Jerusalem, Israel; 2 Department of Inorganic and Analytical Chemistry, The Hebrew University, Jerusalem, Israel; 3 Institut National de la Santé et de la Recherche Médicale, U768, Paris, France; 4 Université Paris-Descartes, Faculté de Médecine René Descartes, Site Necker, IFR 94, Paris, France; 5 Assistance Publique-Hôpitaux de Paris, Hôpital Necker Enfants Malades, Service d'Immunologie et d'Hématologie Pédiatrique, Paris, France; University of Minnesota, United States of America

## Abstract

Non-Homologous End Joining (NHEJ) is one of the two major pathways of DNA Double Strand Breaks (DSBs) repair. Mutations in human NHEJ genes can lead to immunodeficiency due to its role in V(D)J recombination in the immune system. In addition, most patients carrying mutations in NHEJ genes display developmental anomalies which are likely the result of a general defect in repair of endogenously induced DSBs such as those arising during normal DNA replication. Cernunnos/XLF is a recently identified NHEJ gene which is mutated in immunodeficiency with microcephaly patients. Here we aimed to investigate whether Cernunnos/XLF mutations disrupt the ability of patient cells to respond to replication stress conditions. Our results demonstrate that Cernunnos/XLF mutated cells and cells downregulated for Cernunnos/XLF have increased sensitivity to conditions which perturb DNA replication. In addition, under replication stress, these cells exhibit impaired DSB repair and increased accumulation of cells in G2/M. Moreover Cernunnos/XLF mutated and down regulated cells display greater chromosomal instability, particularly at fragile sites, under replication stress conditions. These results provide evidence for the role of Cernunnos/XLF in repair of DSBs and maintenance of genomic stability under replication stress conditions. This is the first study of a NHEJ syndrome showing association with impaired cellular response to replication stress conditions. These findings may be related to the clinical features in these patients which are not due to the V(D)J recombination defect. Additionally, in light of the emerging important role of replication stress in the early stages of cancer development, our findings may provide a mechanism for the role of NHEJ in preventing tumorigenesis.

## Introduction

DNA Double Strand Breaks (DSBs) are one of the most hazardous forms of DNA damage to the integrity of the genome. DSBs arise from exogenous sources such as Ionizing Radiation (IR) but can also be endogenously induced by the production of reactive oxygen species or during normal DNA replication [1]. A failure to repair DSBs could lead to cell death or to chromosomal rearrangements [1]. There are two main DSB repair pathways, the Homologous Recombination (HR) and the Non-Homologous End Joining (NHEJ) which are conserved in eukaryotes from yeast to human [1], [2]. The NHEJ repair process begins with binding of the DNA ends by the Ku70/Ku80 heterodimer and recruitment of DNA-PK catalytic subunit (DNA-PKcs). This is followed by processing of the DNA ends which involves Artemis and/or other DNA processing enzymes. The final step is the ligation of the two DNA ends by DNA Ligase IV, which acts in a complex together with XRCC4 and Cernunnos/XLF that stabilize it and stimulate its activity (for review see[3], [4]).

Cernnunos/XLF is a recently discovered NHEJ core factor which acts as part of the Ligase IV/XRCC4 complex and bares structural similarity to XRCC4 [5], [6]. Cernnunos/XLF has been shown *in vitro* to stimulate the ligation process by the Ligase IV/XRCC4 complex [7], [8]. Cernnunos/XLF deficient mouse embryonic fibroblasts are highly sensitive to IR and show increased chromosomal abnormalities implicating the importance of Cernnunos/XLF in preventing genomic instability under normal growth conditions [9].

Mutations in the human Cernnunos/XLF gene cause combined immunodeficiency, due to a defect in V(D)J recombination which leads to decreased numbers of mature T- and B- lymphocytes [5], [10]. Fibroblasts from patients carrying Cernunnos/XLF mutations are radio-sensitive and exhibit impaired DSB repair following IR or radiomimetic drug treatment [5], [6]. These results demonstrate that Cernunnos/XLF mutations disrupt not only V(D)J recombination but also the repair of IR- induced DSBs. Patients carrying Cernnunos/XLF mutations also display clinical features which are not related to the immune system such as growth retardation, microcephaly, various dysmorphic features and malformations. In addition these patients show chromosomal rearrangements, indicating difficulty in maintaining genomic stability. All these phenotypes might indicate a defect in repair of DSBs resulting from intrinsic sources such as DNA replication. However, the role of Cernnunos/XLF in the repair of replication–induced DSBs has not been studied, nor the effect of Cernnunos/XLF mutations on the response to replication stress conditions.

Replication-induced DSBs are thought to be repaired primarily through the HR pathway which uses a homologous chromatid or chromosome for the repair [2] and therefore can readily repair DSBs during DNA replication as the two sister chromatids are localized in close proximity [11]. Nevertheless, the NHEJ repair pathway also takes part in the repair of replication-induced DSBs [12], [13]. NHEJ deficient mammalian cells were shown to be more sensitive to various replication stress inducing agents [12]–[14]. Moreover, we recently determined that the DNA-PKcs and Ligase IV NHEJ factors are important for maintenance of common fragile site stability under replication stress conditions [15]. Common fragile sites are the loci in the genome that are prone to DSB formation under conditions of replication stress and are involved in chromosomal rearrangements in tumors [16], [17]. Therefore, we hypothesized that since Cernunnos/XLF has a role in NHEJ which is not confined to V(D)J recombination, Cernunnos/XLF mutations can lead to sensitivity to replication stress and to increased fragile site instability

We tested this hypothesis by analyzing the cellular response to replication stress conditions of cells from patients with immunodeficiency and microcephaly, caused by nonsense or framshift mutations in the Cernnunos/XLF gene and in cells down regulated for cells downregulated for Cernunnos/XLF by siRNA. We demonstrate that the mutated and down regulated cells are sensitive to replication stress conditions. Under mild replication stress, the mutated as well as the downregulated Cernunnos/XLF cells exhibit impaired DSB repair. We further show that under replication stress conditions Cernnunos/XLF mutated cells display increased chromosomal instability at common fragile sites. This effect is most likely due to defective repair of replication-induced DSBs and not due to perturbed replication since we found that the replication rate in Cernunnos/XLF mutated cells is normal. These results provide evidence for the importance of Cernunnos/XLF in DSB repair, maintenance of genomic stability and survival under replication stress conditions. Hence, we conclude that Cernunnos/XLF mutations in immunodeficiency patients disrupt the ability of cells to respond appropriately to replication stress conditions and therefore may jeopardize the cells when they are hyperproliferating during embryonic development or in early stages of tumor development.

## Results

### Cernunnos/XLF deficiency causes increased sensitivity to replication stress

**Figure 1:**
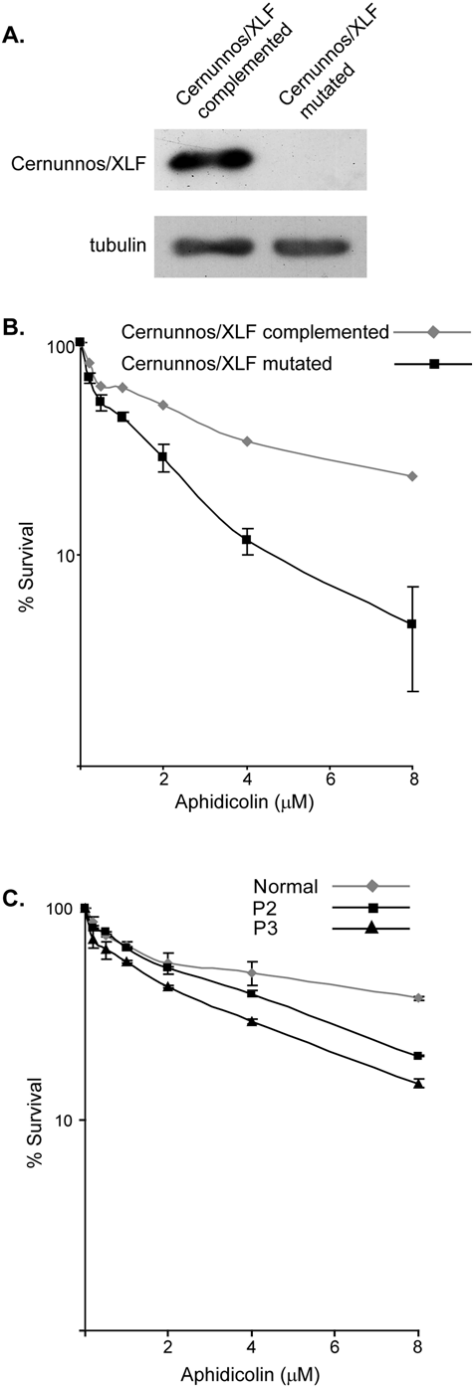
Cernunnos/XLF mutation leads to increased sensitivity to aphidicolin treatment. A. Western blot probed with an anti Cernunnos/XLF antibody in U2OS cells MRC5 Cells, Cernunnos/XLF mutated and Cernunnos/XLF complemented cells. The antibody recognizes an epitope at the C-terminus of the protein which is found beyond the premature stop-codon created by the nonsense mutation in the Cernunnos/XLF mutated cells. The Cernunnos/XLF band is slightly higher in the Cernunnos/XLF complemented cells compared with other cells (U2OS and MRC5) since it is myc-tagged. B. Cernunnos/XLF mutated and complemented cells were treated with the indicated aphidicolin concentrations for 48 hours. Survival was assessed by crystal violet staining. Data points represent mean and standard errors of two independent experiments. The standard errors in Cernunnos/XLF complemented cells are very small. C. Primary fibroblasts from P2, P3 and control primary fibroblasts were treated with the indicated aphidicolin concentrations for 144 hours. Survival was assessed by crystal violet staining. Data points represent mean and standard errors of two independent experiments.

In order to test our hypothesis that Cernunnos/XLF mutations disrupt the cellular response to replication stress we used a cell line derived from a patient carrying a homozygous nonsense mutation in the Cernunnos/XLF gene [5], which leads to complete absence of the full length Cernunnos/XLF protein (Fig 1A). As a control we used the same cells complemented with the wild type gene (Fig 1A).

We first analyzed the survival of Cernunnos/XLF mutated and complemented cells following treatment with aphidicolin, an inhibitor of DNA polymerase α, δ and ε [18], [19]. We found that Cernunnos/XLF mutated cells are markedly more sensitive to aphidicolin than their complemented counterparts (Fig 1B). This was evident in low as well as high aphidicolin concentrations. A similar experiment was performed on primary fibroblasts of the same patient (P2) and on primary fibroblasts from another patient (P3) carrying homozygous deletion G267 (K69fs) [5]. As a control, we used primary fibroblasts derived from a T-B- SCID patient with a mutation in the Rag1 gene and normal Cernunnos/XLF alleles. Rag 1 is exclusively expressed in immature lymphocytes therefore in these cells it is not expressed and has no effect on the analyzed phenotypes. As can be seen in Fig 1C, the P2 and P3 primary cells are more sensitive to aphidiclin than the primay cells with normal Cernunnos/XLF alleles, indicating that the sensitivity to aphidicolin of the transformed cells is caused by the lack of functional Cernunnos/XLF and not due to the transformation itself.

Together, our results indicate that Cernunnos/XLF is required for cellular survival under conditions which perturb elongation of DNA replication and that Cernunnos/XLF mutations found in patients, disrutpt the cellular ability to cope with these conditions.

### Cernunnos/XLF deficiency causes defect in the repair of replication-induced DSBs

Cernunnos/XLF is a core factor of NHEJ hence we investigated whether increased sensitivity to replication stress could be due to impaired DSB repair. For this we analyzed the phosphorylation of histone H2AX on Ser139 following aphidicolin treatment. Histone H2AX undergoes phosphorylation in response to DSBs originating from diverse origins, including replication fork collapse [20]–[22]. The degree of histone H2AX phosphorylation gives a fairly accurate assessment of the extent of DSB repair [23].

**Figure 2:**
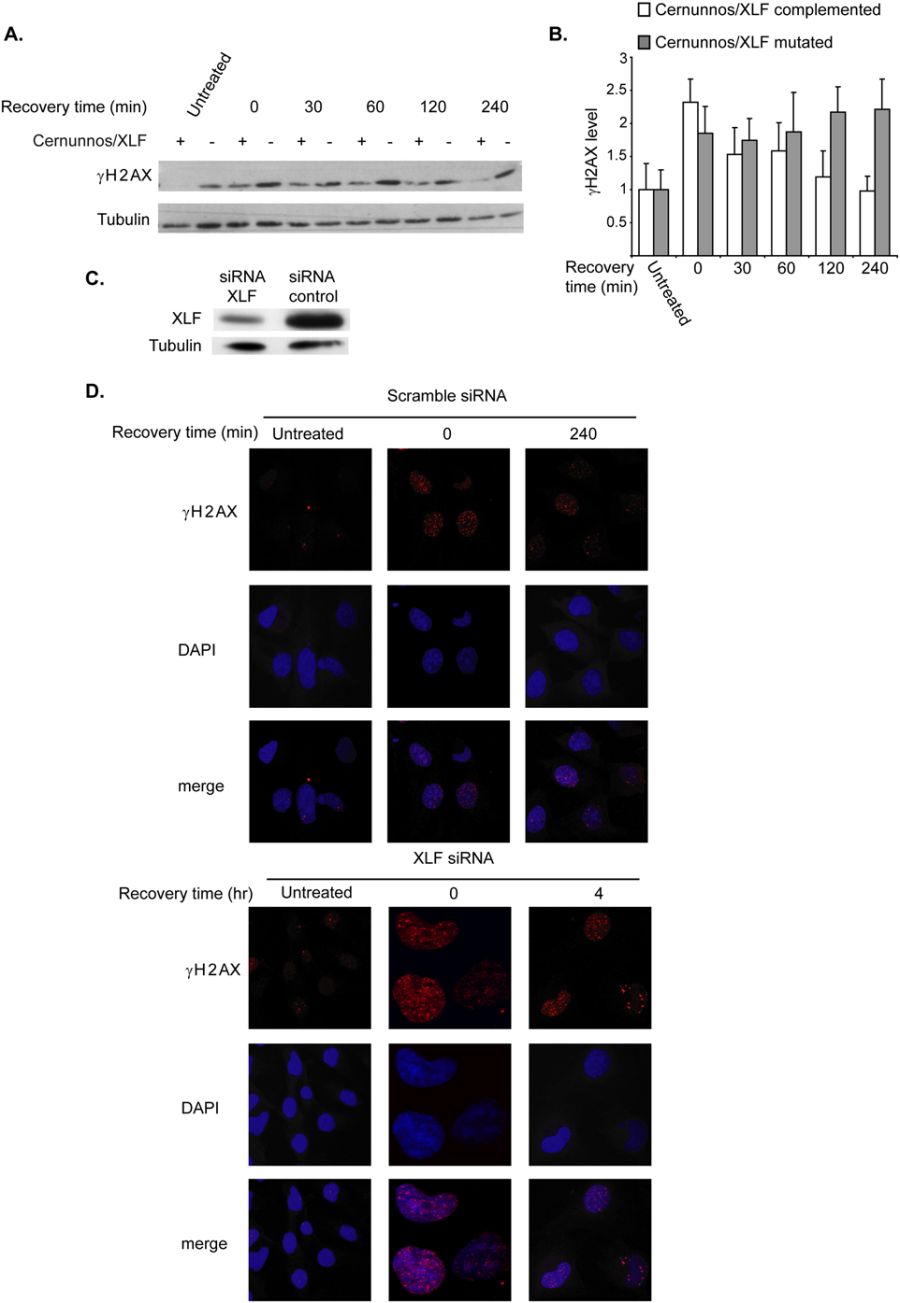
Cernunnos/XLF mutated or downregulated cells exhibit impaired repair of DSBs following treatment with low aphidicolin concentrations. A. Cernunnos/XLF mutated and complemented cells were treated with 0.5 µM aphidicolin for 24 hours and were then transferred to regular growth media for 0–240 minutes. Protein Extracts were prepared at the indicated times after transfer to regular media and analyzed by immunoblotting with anti- γH2AX antibodies (α-γH2AX). Untreated cells were analyzed as control. B. Quantification of γH2AX levels in Cernunnos/XLF mutated and complemented cells following 0.5 µM aphidicolin treatment for 24 hours and different recovery times. γH2AX level was normalized to tubulin control and to untreated cells. Bars represent mean and standard error of three independent experiments C. Western blot probed with anti- Cernunnos/XLF antibody in BJ cells transfected with siRNA against Cernunnos/XLF. Transfection with a nonspecic siRNA was analyed as a control. Equal loading was confirmed by probing with an anti-Tubulin antibody. D. BJ cells transfected with siRNA against Cernunnos/XLF or non-specific control siRNA following 0.5 µM aphidicolin treatment for 24 hours and at a recovery time of 240 minutes. Cells were fixed and stained with anti γH2AX antibody.

Western blot analysis using an antibody directed against the phosphorylated form of histone H2AX (γH2AX) was performed in the Cernunnos/XLF mutated and complemented cell lines, treated for 24 hours with low aphidicolin concentration, under conditions that do not arrest DNA replication, and allowed to recover in regular media for different times (Fig 2A, B). In both Cernunnos/XLF mutated and complemented cells the level of γH2AX increased significantly following the treatment (p<0.05) and stayed high at the first two hours of recovery (Fig 2B). After 2 and 4 hours of recovery in Cernunnos/XLF complemented cells γH2AX levels were significantly reduced (p<0.05) while in the Cernunnos/XLF mutated cells γH2AX level was still high, indicating an impaired repair of replication- induced DSBs in the absence of Cernunnos/XLF. Interestingly, in untreated cells the phosphorylation level of histone H2AX was higher in Cernunnos/XLF mutated cells than in Cernunnos/XLF complemented cells (Fig 2A). This could indicate that Cernunnos/XLF mutations lead to a defect in repair of spontaneous DSBs even under normal growth conditions. An impaired repair of replication-induced DSBs in the absence of Cernunnos/XLF was shown also by γH2AX foci analysis, which represent sites of DNA damage (Rogakou 1998,1999). The analysis was performed on BJ cells, following 24 hours treatment with aphidicolin, in which the Cernunnos/XLF protein was knocked down, using siRNA against Cernunnos/XLF (Fig 2C, 2D). No increase in cell death was observed in Cernunnos/XLF mutated cells after 24 hour aphidicolin treatment compared to their complemented counterparts (data not shown), indicating that cell death can not explain their higher γH2AX levels.

**Figure 3:**
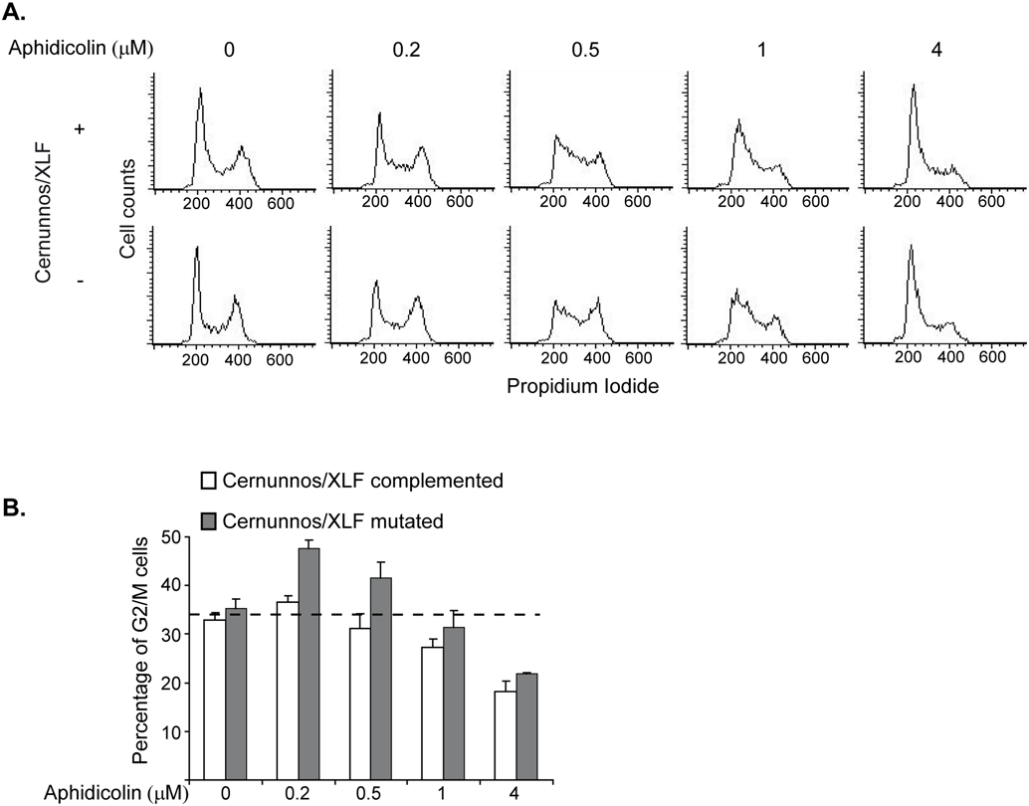
Cernunnos/XLF mutated cells exhibit Increased accumulation of cells in G2/M following treatment with low aphidicolin concentrations. A. Cernunnos/XLF mutated and complemented cells were treated with the indicated aphidicolin concentrations for 24 hours, fixed, stained with propidium iodide and analyzed by FACS for DNA content. B. Quantification of the percentage of cells in G2/M following aphidicolin treatment. Bars represent mean and standard error of three independent experiments. The dashed line represents the approximate percentage of cells in G2/M in untreated cells.

A delay in repair of replication–induced DSBs is expected to affect cell cycle progression due to checkpoint activation, therefore we analyzed the cell cycle of aphidicolin-treated cells (Fig 3A). Aphidicolin can cause a change in cell cycle progression either directly through the inhibition of DNA polymerases or indirectly through activation of checkpoints in response to induced DNA damage. The direct effect could cause lengthening of S-phase in low concentrations, or arrest of cells along S-phase in high concentrations. The indirect effect would lead to accumulation of cells in S or G2/M due to activation of checkpoints. The cell cycle distribution of Cernunnos/XLF mutated and complemented cell lines was very similar in untreated cells (Fig 3A, B) despite complete lack of NHEJ in the mutated cells [5]. In the lower aphidicolin concentrations (0.2 µM and 0.5 µM), the percentage of G2/M cells was significantly higher in Cernunnos/XLF mutated cells compared with Cernunnos/XLF complemented cells (p<0.05, Fig 3A, B), indicating higher levels of DNA damage in Cernunnos/XLF mutated cells compared to their complemented counterparts under the same conditions. In the higher aphidicolin concentrations (1 µM and 4 µM) most of the cells are stuck in the beginning of S-phase due to strong inhibition of DNA polymerase, therefore the difference in the percentage of G2/M cells seen between mutated and complemented cells is small and insignificant. Thus, these results provide additional support that Cernunnos/XLF deficiency leads to impaired repair of replication-induced DSBs.

**Figure 4:**
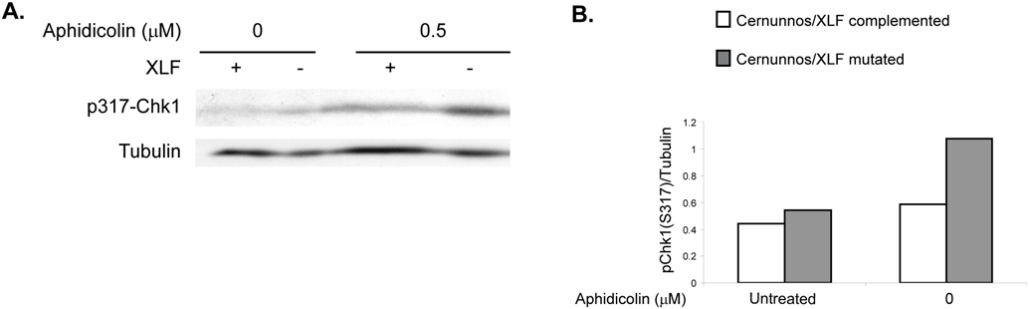
The Cernunnos/XLF mutation lead to an increased activation of Chk1 in response to replication stress. A. Cernunnos/XLF mutated and complemented cells were treated with 0.5 µM aphidicolin for 24 hours. Protein extracts were prepared and analyzed by immunoblotting with anti- pChk1(S317) antibodies. Untreated cells were analyzed as control. B. Quantification of pChk1(S317) levels in Cernunnos/XLF mutated and complemented cells following 0.5 µM aphidicolin treatment for 24 hours. pChk1(S317) level was normalized to Tubulin control.

Replication-induced DNA damage was shown to activate the cell cycle checkpoint protein, Chk1 [24]. It was recently demonstrated that low concentrations of aphidicolin which do not arrest DNA replication, activate the cell cycle checkpoint protein, Chk1 [25]. Hence, we further analyzed the activation of Chk1 in Cernunnos/XLF mutated and complemented cell lines which were treated for 24 h with 0.5 µM aphidicolin, using an antibody against phosphorylated Chk1 (Fig 4). In the Cernunnos/XLF mutated cells the level of phosphorylated Chk1 increased following the treatment, indicating the Chk1 is activated following the replication stress conferred by aphidicolin. However, the results clearly show a higher activation in Cernunnos/XLF mutated and down regulated cells compared with the complemented cells (Fig 4). These results indicate that in Cernunnos/XLF mutated cells, the inability to efficiently repair replication-induced DSBs leads to increased Chk1 activation.

### Increased replication stress induced fragile site instability in Cernunnos/XLF mutated cells

**Figure 5:**
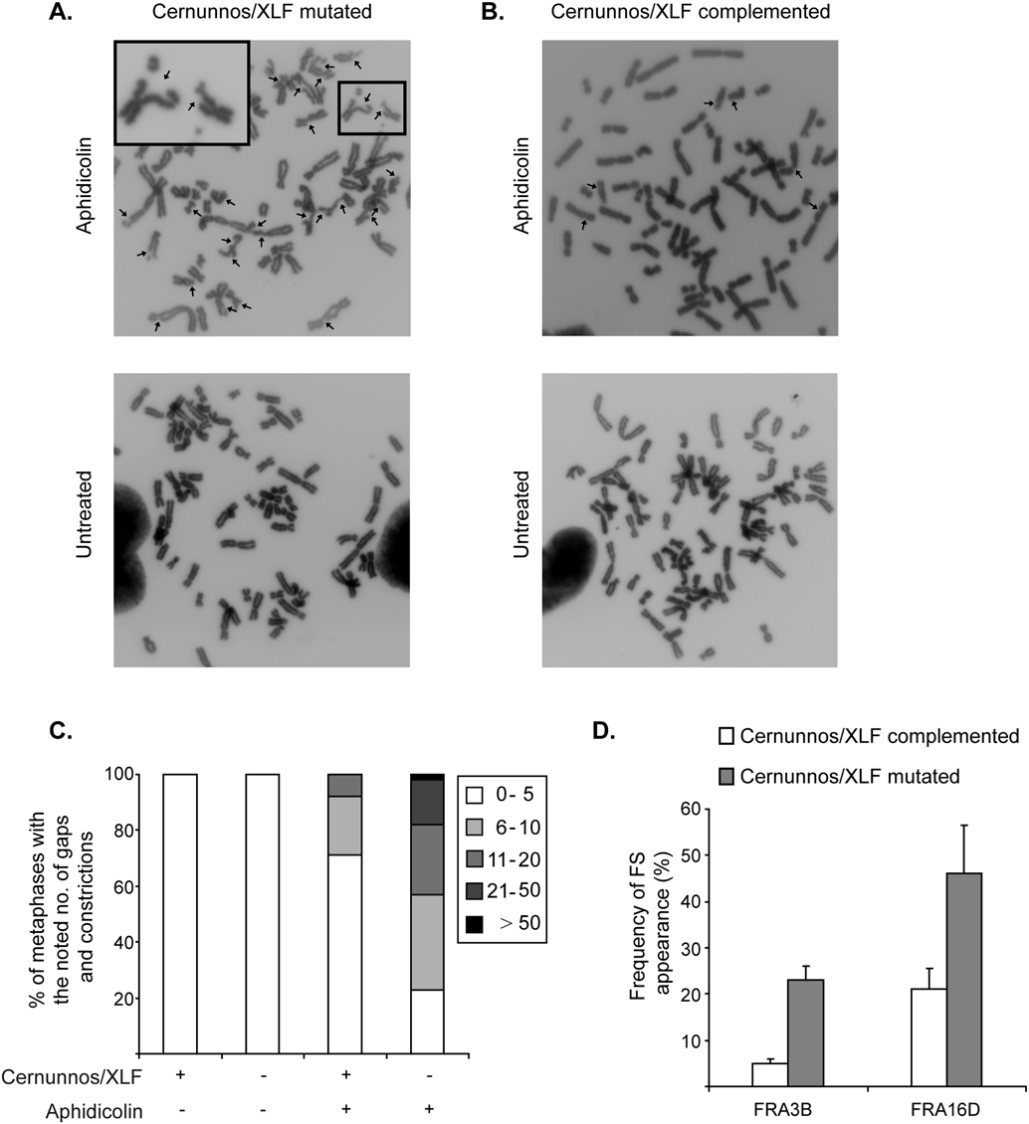
Cernunnos/XLF mutation leads to increased common fragile site instability. A. Examples of metaphases from Cernunnos/XLF mutated cells. A cell treated with 0.2 µM aphidicolin for 24 hours, showing a high number of gaps and constrictions (n = 28). The box in the top left is a magnification of the area marked in the picture. Arrows mark gaps and constrictions (top panel). An untreated cell showing no gaps and constrictions (bottom panel). B. Examples of metaphases from Cernunnos/XLF complemented cells. A cell treated with 0.2 µM aphidicolin for 24 hours, showing an average number of gaps and constrictions (n = 6). Arrows mark gaps and constrictions (top panel). An untreated cell showing no gaps and constrictions (bottom panel). C. Number of gaps and constrictions per metaphase in Cernunnos/XLF mutated and complemented cells with or without treatment with 0.2 µM aphidicolin for 24 hours. The data presented is based on two independent experiments. For each experiment, at least 50 metaphases for each condition were analyzed. D. Frequency of chromosomal gaps and constrictions at common fragile sites (FS) FRA3B and FRA16D following treatment with 0.2 µM aphidicolin for 24 hours in Cernunnos/XLF mutated and complemented cells. Bars represent mean and standard error of two independent experiments. For each experiment, at least 50 hybridizations were analyzed.

Common fragile sites are genomic loci which are specifically unstable and appear as gaps and constrictions on metaphase chromosomes from cells grown under conditions of replication stress [26]. Previous studies have demonstrated the involvement of proteins from the DNA damage response pathway in maintenance of common fragile site stability. These include ATR, ATM, CHK1, BRCA1, SMC1, FANCD2 and WRN [27], [28] and reviewed in [29]). Importantly, both HR and NHEJ DSB repair pathways were shown to be important for the maintenance of common fragile site stability under replication stress conditions [15]. A number of human diseases resulting from mutations in DNA damage response genes were shown to be associated with increased instability at common fragile sites [28], [30], [31] however, none of the diseases caused by mutations in the NHEJ factors were studied. Therefore we investigated whether Cernunnos/XLF mutations can lead to increased fragile site instability. For this we analyzed formation of gaps and constrictions in metaphases from Cernunnos/XLF mutated and complemented cell lines that were treated with aphidicolin, as well as in cells down regulated for Cernunnos/XLF expression using siRNA. Following a 24 hours treatment with low aphidicolin concentration, under conditions that do not arrest DNA replication, cells carrying a Cernunnos/XLF mutation showed a significantly higher level of gaps and constrictions (∼3 fold, Fig. 5A,C), compared with their complemented counterparts (p<0.001) (Fig. 5B,C). In the Cernunnos/XLF mutated cells most of the metaphases (>75%) showed >5 gaps and constrictions, including over 15% of the metaphases with a high number of gaps and constrictions (>20), which were not seen in the complemented cells (Fig. 5A, C).

It is known that most chromosomal gaps and constrictions following aphidicolin treatment occur at fragile sites [26]. To verify that the increase in gaps and constrictions, in Cernunnos/XLF mutated cells is at common fragile sites, we analyzed the cloned common fragile sites FRA3B and FRA16D, using fluorescent *in-situ* hybridization (FISH) with specific probes for these sites. Cernunnos/XLF deficiency led to 4.5-fold (p<0.001) and 2-fold (p<0.001) increase in the occurrence of gaps and constrictions at FRA3B and FRA16D respectively, under conditions of replication stress, compared with complemented cells (Fig. 5D). The frequency of gaps and constrictions in untreated cells was extremely low (0.5 FS/ metaphase) and no increase in their frequency was detected in Cernunnos/XLF mutated cells compared to complemented cells (Fig. 5C). Therefore analysis of gaps and constrictions at specific fragile sites was not further analyzed, in untreated cells.

### Normal replication rate in Cernunnos/XLF mutated cells

**Figure 6:**
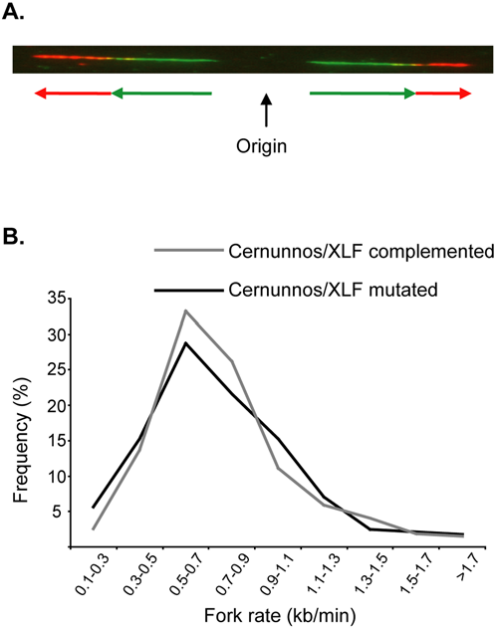
Cernunnos/XLF mutation does not affect replication rate. A. Example of a DNA fiber showing the labeled IdU pulse (green signals and arrows) and the labeled CldU pulse (red signals and arrows). The estimated origin of replication is marked by a black arrow. B. Distribution of fork rate in untreated Cernunnos/XLF mutated and complemented cells. The data presented is based on two independent experiments. In each experiment at least 50 molecules each containing at least two replication signals were analyzed for each cell line.

As mentioned above replication-induced DSBs are repaired by both HR and NHEJ DSB repair pathways. A recent report by Daboussi et al. showed that deficiency in HR factors in mammalian cells results in slower replication fork progression [32]. Here we examined a possible effect of Cernunnos/XLF mutation on replication fork progression. For this we analyzed replication rate in Cernunnos/XLF mutated and complemented cell lines by DNA combing [33]. Nascent DNA was labeled in the cells by an IododeoxyUridine (IdU) pulse followed by a ChlorodeoxyUridine (CldU) pulse. Fork rate was calculated by measuring replication signals visualized following fluorescent detection of IdU and CldU on single DNA molecules which were spread on silanized microscope slides (Fig. 6A). Average replication rate and the distribution of fork rate were similar in Cernunnos/XLF mutated and complemented cells (0.76+/−0.35 kb/min and 0.76+/−0.31 kb/min respectively and Fig. 6B). These results indicate that the absence of Cernunnos/XLF does not affect replication fork progression.

## Discussion

Here we provide evidence for the importance of Cernunnos/XLF for DSB repair, maintenance of genomic stability and survival under replication stress conditions. We found that cells harboring a nonsense mutation in the Cernunnos/XLF gene from human patients with immunodeficiency with microcephaly, exhibit decreased survival following aphidicolin treatment, indicating increased sensitivity to replication stress conditions (Fig 1B). Since Cernunnos/XLF is a NHEJ factor, we speculated that the increased cell death seen under replication stress conditions is probably due to a defect in repair of replication-induced DSBs. Indeed our results show that Cernunnos/XLF mutated cells exhibit impaired DSB repair process and increased accumulation of cells in G2/M following aphidicolin treatment (Fig 3). Moreover, Cernunnos/XLF mutated cells display increased chromosomal instability at common fragile sites following mild replication stress conditions (Fig 5). This effect of Cernunnos/XLF mutation on the stability of fragile sites was not the result of perturbed replication progression as shown in Figure 6.

Several patients have been described in the literature harboring mutations in the NHEJ factors Ligase IV, Artemis or Cernunnos/XLF [5], [34]–[38]. In the majority of the patients, mutations in any of these genes leads to immunodeficiency, indicating the immediate effect of mutations in NHEJ on V(D)J recombination. In many of the cases, including those harboring mutations in Cernunnos/XLF, patients are born with developmental anomalies, such as microcephaly and growth retardation, and show chromosomal instability. These phenotypes are probably not related to the V(D)J recombination defect in the immune system, but rather likely result from a general inability to repair spontaneous DSBs occurring during embryonic development throughout the body. One major source of spontaneous DSBs is the DNA replication process. Indeed cells carrying mutations in DNA-PK were shown to be deficient in repair of replication-induced DSBs [39]. Our results provide the first evidence that a disease caused by mutations in NHEJ genes is associated with defective response to conditions which perturb DNA replication. The developmental anomalies seen in patients carrying mutations in NHEJ genes may result from inappropriate DSB repair and increased chromosomal instability at times of replicative stress which may occur when cells are hyperproliferating during development [40].

Studies in humans and mice raised the possibility that decreased NHEJ activity plays a role in carcinogenesis. In both mice and humans, NHEJ deficiency is associated with tumor development [35], [37], [41]–[43]. Genetic analyses in several human malignancies (germ cell tumors and high grade breast cancers among others) revealed that over 30% of the tumors had deletions in the distal portion of chromosome 13 harboring the Ligase IV gene (Helsinki University CGH database, [44], [45]). In addition, genotypic polymorphisms in NHEJ genes (XRCC4, Ligase IV and Ku 70) have been associated with increased risk to develop breast cancer or glioma [46]–[48]. Since Cernunnos/XLF was only recently identified, its association with cancer risk, in humans or mice, was not yet studied. Moreover, cancer has not been reported in patients carrying Cernunnos/XLF mutations but only few patients were as of yet reported, of whom several already died from fatal infections and the others are still young.

Recent studies demonstrated that the immediate event following oncogene overexpression is hyperproliferation, associated with replication perturbation and formation of replication-induced DSBs [49], [50]. The barrier to tumorigenesis is exerted by the DNA damage response [51], [52]. Therefore damage response factors, involved in the cellular response to replication-induced DSBs, are likely to act as caretakers of the genome during tumor development. Here we show that cells from patients carrying mutations in the NHEJ gene Cernunnos/XLF exhibit sensitivity to replication stress and impaired repair of replication-induced DSBs. In light of the crucial role of replication stress at the early stages of cancer development, our results suggest that the impaired response to replication stress could be the mechanism by which defective NHEJ contributes to human carcinogenesis. Furthermore, our results imply that Cernunnos/XLF acts as a caretaker of genome stability and can be a potential factor in the prevention of tumorigenesis.

The results from our fragile site analysis indicate that Cernunnos/XLF is crucial for the maintenance of fragile site stability under conditions which perturb DNA replication but not under normal growth conditions (Fig 5C). This is in agreement with our previous results showing that down-regulation of DNA-PKcs or Ligase IV by RNAi increased fragile site instability only under replication stress [15]. It is interesting to note that under normal growth conditions Cernunnos/XLF mutated cells exhibit higher H2AX phosphorylation level compared to the complemented cells, indicating unrepaired spontaneous DSBs in Cernunnos/XLF mutated cells. Nevertheless, these DSBs are probably repaired eventually, perhaps due to increased activity of other repair pathways or prolonged arrest in cell cycle checkpoints allowing maintenance of fragile site stability. Importantly, Rad51, a component of HR, has a role in maintaining fragile site stability under both replication stress and normal growth conditions [15]. These results can suggest that the defect in repair of replication-induced DSBs caused by NHEJ deficiency could be compensated by HR, but not vice versa. Indeed, DNA-PKcs deficiency was shown to result in increased repair of DSBs by HR [53]. Another possibility is that HR has a role during normal progression of DNA replication while NHEJ is important only when DNA replication is perturbed. In support of the latter, a recent study shows that defects in several HR factors (Rad51, BRCA2 or XRCC2) affect replication fork progression [32], while we found that Cernunnos/XLF deficiency has no effect on DNA replication (Fig. 6). Our results suggest that the negative effect of Cernunnos/XLF mutations on genome stability in immunodeficiency and microcephaly patients would probably be prominent in hyperproliferating cells when DNA replication could be occasionally perturbed.

In summary, the results presented in this study demonstrate that cells from patients carrying mutations in the Cernunnos/XLF NHEJ gene have impaired ability to respond to replication stress. This might explain the developmental anomalies which are not related to V(D)J recombination in patients carrying mutations in NHEJ genes. Moreover, our data suggest that the impaired response to replication stress could be the mechanism by which defective NHEJ contributes to human carcinogenesis.

## Materials and Methods

### Cells, growth conditions and treatment

Cernunnos/XLF mutated cells are SV40T-transformed, telomerase-immortalized fibroblasts from patient P2 as published [5]. Complementation of the Cernunnos/XLF mutated cells was performed by transduction of the cells with pMND-Cernunnos-Myc-IRES-GFP retroviral vector. Cells were grown in RPMI medium supplemented with 10% fetal bovine serum. hTERT BJ1 cell line (CLONTECH) were grown in DMEM supplemented with 10% fetal bovine serum. Aphidicolin treatment was performed by growing cells for the indicated time in RPMI media containing the indicated aphidicolin concentration.

Primary fibroblasts derived from P2 and P3 [5] were grown in RPMI medium supplemented with 16% fetal bovine serum.

### Survival assay

Cernunnos/XLF mutated and complemented cells were plated on 96-well plate at a density of 4000 cells/well and aphidicolin treatment was performed in triplicates for 48 hours. Primary fibroblasts were plated on 96-well plate at a density of 6000 cells/well and aphidicolin treatment was performed in triplicates for 144 hours. This longer aphidicolin treatment was required, since the primary cells grow significantly more slowly than the transformed cells. Afterwards cells were washed with PBS, fixed in 3.7% formaldehyde/PBS and stained for 30 minutes in 0.1% crystal violet. After washing and drying the crystal violet staining, the dye was extracted by 10% acetic acid and the absorbance of each well was measured at 590 nm by a microplate reader (Tecan).

### Western blot

15% and 10% Polyacrylamide gels were used for protein separation and detection of γH2AX and Cernunnos/XLF and phosphorelated S317 Chk1 respectively. The gel was transferred to a nitrocellulose membrane and antibody hybridization and chemiluminescence were performed according to standard procedures. γH2AX was detected with mouse antibodies (Upstate biotechnology). Cernunnos/XLF was detected with rabbit antibodies (Bethyl laboratories) and phosphorelated S317 Chk1 (S-317) was detected with rabbit antibodies (Cell Signaling technology). HRP-conjugated anti-mouse and anti-rabbit secondary antibodies were obtained from Jackson Immunoresearch laboratories.

### Immunofluorescence

Cell were fixed in 3.7% formaldehyde/PBS for 10 min, permeabilized with 0.5% Triton/PBS and blocked with 5% BSA/PBS. The primary antibody used in this study was mouse anti γH2AX (Upstate Biotechnology). Cy3 conjugated secondary antibody was added (Jackson Immunoresearch Laboratories). The DNA was stained with 2 µg/ml. Images was taken with a Bio-Rad confocal microscope.

### Cell cycle analysis

Following aphidicolin treatment, cells were harvested and fixed in 70% chilled Ethanol overnight. Cells were then stained with 0.01 µg/µl propidium iodide and DNA content was analyzed by flow cytometry (Becton Dickinson FACScalibur).

### Chromosomes preparation and fragile site analysis

Cells were harvested after a 40 minutes treatment with 100 ng/ml colchicine followed by a 40 minutes incubation in 0.4% KCl at 37°C and multiple changes of 3∶1 methanol: acetic acid fixative. Cells were dropped onto slides and slides were baked overnight at 37°C before FISH protocol. BAC clones crossing or within fragile sites were used for FISH analysis. BAC 1O12 was used for FRA3B and BAC 264L1 was used for FRA16D. Probes were labeled with digoxigenin (DIG)-11-dUTP (Roche) by nick translation. DIG-labeled probes were detected with fluorescein isothiocyanate (FITC)-conjugated sheep anti-DIG specific antibodies (Roche) and the signal was amplified using donkey anti-sheep Cy2 antibodies (Jackson Immunoresearch laboratories). DNA was stained with propidium iodide. FISH on metaphase chromosomes was performed as previously described [54].

Gaps and constrictions at fragile sites were analyzed using a Nikon fluorescent microscope. For total gaps and constrictions, at least 100 metaphases for each condition were analyzed. For analysis of specific fragile sites, at least 100 hybridizations were analyzed.

### Replication analysis

Cells were pulse labeled for 30 min by replacing the normal medium with pre-warmed medium containing 100 µM iododeoxyuridine (IdU).At the end of the first labeling period, cells were washed twice with warm phosphate-buffered saline (PBS) and then pulse labeled once more for 30 min with medium containing 100 µM chlorodeoxyuridine (CldU). Cells were then harvested and genomic DNA was extracted and combed as previously described [55]. Briefly, cells were resuspended in PBS at a concentration of 10^7^ cells/ml, mixed with an equal volume of 1% low-melting point agarose and immediately poured into 100 µl molds. Before use, DNA containing agarose blocks were washed in TE buffer and melted in MES buffer followed by digestion of agarose by β-agarase. Following combing of the DNA on silanized cover slips, DNA was denatured in 1 M NaOH and replication signals were detected by indirect immunofluorescence. The primary antibodies used were mouse anti-BrdU (for detection of IdU, Becton Dickinson) and rat anti-CldU (Novus Biologicals). The secondary antibodies used were alexa fluor 488 goat anti-mouse and alexa fluor 594 goat anti-rat (invitrogen). Slides were mounted in Slowfade light antifade reagent (molecular probes) and analyzed using an Olympus IX81 inverted fluorescence microscope equipped with an Andor IXON-885 electron-multiplying CCD camera using the Andor IQ software (Andor Bioimaging Division, Morrisville, NC). For replication rate analysis at least a 100 molecules, each containing at least two replication signals were analyzed for each cell line.

### Statistical analysis

For comparison of total gaps and constrictions the Kolmogorov-Smirnov two-sample test was used. For comparison of gaps and constrictions at specific fragile sites Fisher's test was used. For statistical analysis of γH2AX level and percentage of G2/M cells, t-test was used.

### RNA interference

The siRNA sequence directed against Cernunnos/XLF was constructed by IDT (Coralville, IA, USA) sequence: 5′-CGCUGAUUCGAGAUCGAUUGATT-3′

Nonspecific control oligo (Dharmacon, Lafayette, CO, USA) contains 52% GC content similar to the GC content in the specific oligo. Oligofectamine (Invitrogen, Carlsbad, CA, USA) was used for transfection of these RNA oligos into Hela and BJ cells.
